# Supplementary figures and images for: Aurora kinase A/AURKA functionally interacts with the mitochondrial ATP synthase to regulate energy metabolism and cell death
Source: Cell Death Discov. 2023 Jun 29;9:203. doi: 10.1038/s41420-023-01501-2 (PMC10310848; doi:10.1038/s41420-023-01501-2)

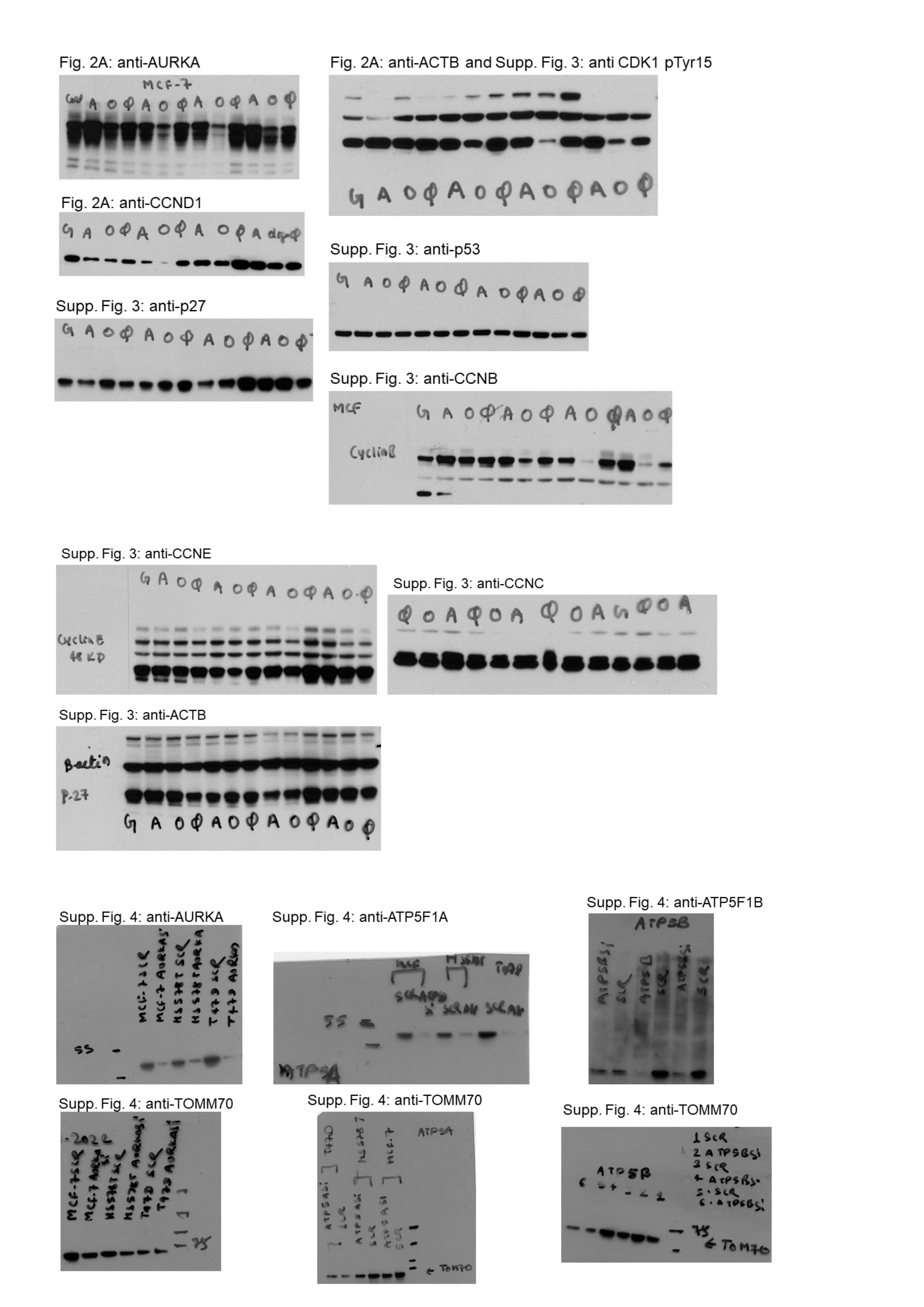

Supplement: Supplementary file 2 — original data files [file 41420_2023_1501_MOESM2_ESM.tif]
